# Genome-wide genetic characterization and selection signatures in Anatolian Merino sheep

**DOI:** 10.5194/aab-68-161-2025

**Published:** 2025-02-26

**Authors:** Taki Karsli

**Affiliations:** 1 Department of Animal Science, Faculty of Agriculture, Eskişehir Osmangazi University, Eskişehir, 26160, Türkiye

## Abstract

Molecular characterization and identification of selection signals at the genome-wide level facilitate the enhancement of ongoing conservation and selection studies in farm animals. This study aimed to reveal genomic diversity and selection signatures in Anatolian Merino sheep via 351 539 bi-allelic single-nucleotide polymorphisms (SNPs) obtained from the double-digest restriction-site-associated DNA sequencing (ddRADseq) technique. Genetic variability parameters such as minor-allele frequency (MAF), nucleotide diversity ( π ), observed heterozygosity ( $H_{O}$ ), and expected heterozygosity ( $H_{E}$ ) were estimated to be 0.340, 0.235, 0.258, and 0.235, respectively, while the inbreeding coefficient was 0.027 based on runs of homozygosity (ROH). A decreasing trend was detected in the effective population size of Anatolian Merino sheep, the current population of which turned out to be descendants of an ancestral population covering 2500 individuals 400 generations ago. Significant selection signals were detected in 464 SNPs within 14 genomic intervals via the ROH approach, whereas 259 SNPs were categorized into 79 genomic intervals by integrated haplotype score (iHS) statistics. A total of 37 and 72 protein-coding genes overlapped with detected genomic intervals in ROH and iHS approaches, respectively. A survey of a sheep QTL database confirmed that selection signals covered 66 QTL-associated SNPs. A large part of the protein-coding genes under selection pressure were mainly associated with milk production (*ROBO1*, *FSIP2*, *COBLL1*, *PTPN12*, *GSAP*, *CCDC146*, *FGL2*, *FAM185A*, *FBXL13*, *LOXL2*, *R3HCC1*, *CHMP7*, *RHOBTB2*, *PEBP4*, *SRGAP3*, and *RAD18*) and udder morphology (*SYT1*, *GFPT2*, *MAPK9*, and *RASGEF1C*), while numerous genes turned out to have effects on total muscle area (*PCDH7*), bone density (*SDK2*), carcass traits (*MBL2*), fecal egg count (*LGSN*), and immunoglobulin A level in blood circulation (*RFXAP*, *SERTM1*, *BEND7*, *PRPF18*, *FRMD4A*, and *GRAMD1B*). The results of this study confirm that high-density next-generation sequencing (NGS) data could be utilized to characterize local sheep breeds to shape conservation programs and shed light on the past breeding practices of the populations whose phenotypic records are absent.

## 1 Introduction

Thanks to their higher adaptability, small ruminants such as goats and sheep are reared to produce milk and meat across diverse climatic zones under different breeding systems (Demir et al., 2022). In Türkiye, grassland-based rearing not only allows for efficiently utilizing lands which are not proper for crop production but also supports the incomes of smallholder farmers in rural areas in order to sustain the production of animal-derived protein resources in a traditional way (Demir, 2024a). Sheep rearing is mainly centered on native Turkish breeds in the country, covering 91 % of 45 million heads in total (Karsli, 2024), while the remaining breeds are believed to be Merino and other crossbreeds. After being introduced to Anatolia in 1839, Merino sheep were subjected to crossbreeding studies with several native Turkish sheep breeds such as Kıvırcık and Akkaraman to improve economically important traits, including wool characteristics (Koyuncu, 2024). Crossbreeding practices between Kıvırcık and Merino have led to the obtainment of a new breed called Karacabey Merino (Koyuncu, 2024), whereas the Anatolian Merino breed was developed via crossbreeding practices between Akkaraman and German Mutton Merino (Kizilaslan et al., 2024). Although Merino-derived breeds were initially reared to produce high-quality fleece for the textile sector during the 1950s, the loss in the economic value of wool production has forced farmers to focus on meat and milk production since then (Behrem and Gül, 2022). It is believed that this shift in breeder preferences has led to the fixation of certain gene regions associated with milk and meat yield in the genome of Anatolian Merino sheep, increasing their frequency. Identifying these gene regions (potential candidate genes) across the whole genome will provide a foundation for future genomic selection studies in this breed

Advances in molecular genetics have facilitated the evaluation of local sheep populations at the genome-wide level in terms of different aspects, including genetic characterization and selection signatures. Genetic characterization is an efficient approach for designing management and conservation programs in local sheep populations (Alnajm et al., 2021). Numerous molecular genotyping methods are available to estimate genetic diversity parameters, such as observed ( $H_{O}$ ) and expected heterozygosity ( $H_{E}$ ), as well as the inbreeding level, which allows one to maintain production in the local sheep population by periodically monitoring variability across the genome. Besides, genome-wide genetic variability gives significant clues with regard to prioritizing populations with regard to which low-variability populations to prioritize in conservation programs (Ben Sassi-Zaidy et al., 2022). Selection signatures, defined as regions with low genetic variability, are valuable indicators for the detection of genomic regions exposed to artificial- and natural-selection pressure. Defined as continuous homozygous segments occurring among a significant number of animals in a certain breed, runs of homozygosity (ROH) is a popular approach to detect genomic regions with lower variability resulting from factors such as genetic drift, bottlenecks, inbreeding, and selection (Peripolli et al., 2017). As articulated by Tiwari et al. (2024), the high proportion of long and short ROH segments indicates long-term and recent selection practices, respectively. The integrated haplotype score (iHS) approach, on the other hand, allows for the detection of recent selection signatures by scanning unusual haplotypes around a single-nucleotide polymorphism (SNP) compared to the whole genome (Voight et al., 2006). These signatures, detected by different approaches, show potential for providing insights into the past breeding experiences (genetic drift, bottleneck effect, geographic expansion, etc.) of local populations and the evolution of sheep genomes throughout history (Kim et al., 2016).

In theory, more reliable results are expected to be obtained with the denser genetic data used in bioinformatic analyses. In practice, on the other hand, using some high-resolution genotyping methods such as arrays of single-nucleotide polymorphisms (SNPs) and whole-genome sequencing (WGS) may not be practical or affordable for smallholders rearing local sheep breeds. Indeed, as articulated by Bilginer et al. (2022), SNP arrays are biased due to being developed based on some reference breeds, which hinders the identification of novel or breed-specific genetic variations in local populations. On the contrary, WGS offers a way to detect almost all genomic variations across the local populations, but it remains expensive, and the sequencing cost per individual is not affordable in many cases (Pedrosa et al., 2021).

Over the last 2 decades, the discovery of next-generation sequencing (NGS) based on genomic library preparation methods has had a ground-breaking effect on the characterization of the local sheep population at a genome-wide level. Of these library preparation methods, double-digest restriction-site-associated DNA sequencing (ddRADseq) allows for the obtainment of millions of short DNA fragments belonging to different individuals which could be sequenced simultaneously (Peterson et al., 2012). The multiplexing process of the ddRADseq method not only decreases the cost of sequencing but also enables scientists to recover adequate genetic data with reduced representations of the genome, which eliminates the complexity of the whole genome. In contrast, ddRADseq includes several laboratory practices (barcode tagging, pooling, enrichment, etc.) which mainly limit the repetition and reproduction of the same genetic data. Besides, numerous sophisticated bioinformatic tools are required in each step (de-multiplexing, quality control, mapping, and filtering) to process raw sequence data into filtered SNP data. ddRADseq-based genetic data have been used for genetic characterization and/or for investigating the signatures of selection in several local species such as cattle (Demir et al., 2023a), sheep (Karsli, 2024), pigs (Vani et al., 2024), and fish (Magris et al., 2022).

By checking the literature, it was detected that the Anatolian Merino breed has been genotyped via microsatellite markers (Öner et al., 2014; Yilmaz et al., 2015; Ameur et al., 2020) for genetic characterization; however, no studies focusing on unraveling selection signatures are available. It is noteworthy that microsatellite markers are insufficient to reveal genome-wide genetic variability and selection signals due to the fact that they represent a small part of the genome, while NGS-based studies have recommended better genetic characterization of native Turkish livestock species (Karsli et al., 2020; Demir, 2024b). In this regard, this study aims, for the first time, to assess the genetic diversity and signatures of selection in Anatolian Merino sheep via genome-wide genetic data obtained from ddRAD sequencing.

## 2 Material and methods

### 2.1 Sampling and DNA isolation

By using pedigree information and oral interviews with animal keepers, a total of 20 unrelated animals belonging to the Anatolian Merino breed were sampled from three herds reared in the province of Eskişehir. In addition, phenotypic identification was conducted to validate the fact that these animals represent the population. For this, 3 mL blood samples were collected from the jugular vein in Vacutainer tubes, with EDTA solution as an anticoagulant. Blood samples were stored at - 20 °C until DNA extraction was performed. By following the manufacturer's guidance, the GeneJET Genomic DNA Purification Kit (Thermo K0721) was deployed to isolate genomic DNA from the blood.

### 2.2 Preparation of genomic libraries and sequencing

As explained in detail by Peterson et al. (2012), the standard ddRADseq protocol was performed to create genomic libraries for the sequencing process. In brief, isolated DNA with the desired quantity and quality was digested with *EcoR*I and *Msp*I restriction enzymes (New England Biolabs). AMPure XP beads (Beckman Coulter) were used to clean digested DNA fragments, which were further supplied with specific molecular barcodes and index primers via T4 DNA ligase. After the enrichment process by polymerase chain reaction (PCR), prepared DNA libraries were subjected to the Illumina NovaSeq 6000 instrument to obtain paired-end reads ( 2×150 base pair).

### 2.3 Raw data processing and variant calling

A methodology used by Demir et al. (2023b) was adopted to process raw short reads into filtered data. To do so, Stacks 2 software (Rochette et al., 2019) was run with supplied barcode and index information to assign each read to individuals. Fastp software (Chen et al., 2018) was run with default parameters for adapter removal and quality trimming. Clean reads were aligned to the *Ovis aries* (ARS-UI_v3.0) genome assembly via default parameters of the Burrows–Wheeler Aligner program (Li and Durbin, 2009). Aligned reads were converted into a Binary Alignment Map (BAM) file format using the SAMtools program (Danecek et al., 2021) with default parameters. The BCFtools (Danecek et al., 2021) pipeline was used to call variants in which all insertion–deletions (InDels) and SNPs not located on autosomal chromosomes were excluded. Bi-allelic SNPs passing specific criteria such as read depth (20 ≤ D ≤ 500) and base quality score ( Q ≥ 20) were kept in the data set. Furthermore, PLINK1.9 software (Chang et al., 2015) was utilized for filtering the data set in terms of SNPs (–maf 0.05 and –geno 0.1) and individuals (–mind 0.1) to call only informative variants with a high genotyping rate. In this study, approximately 12 million short reads were obtained from the sequencing analysis, in which a large part of the reads (98.5 %) were mapped to the reference genome, with a coverage depth of nearly 30 × . After raw data processing and filtering criteria, 351 539 bi-allelic SNPs were recovered across 20 animals.

### 2.4 Bioinformatic analyses

To assess genome-wide genetic variability in Anatolian Merino sheep, several statistical parameters such as minor-allele frequency (MAF), π , $H_{O}$ , and $H_{E}$ were calculated. Of these parameters, MAF, $H_{O}$ , and $H_{E}$ were estimated using PLINK 1.9 software (Chang et al., 2015), whereas VCFtools (Danecek et al., 2011) was deployed to calculate the π value. SNeP v.1.1 software (Barbato et al., 2015) was run with default parameters to assess historical changes in the effective population size ( $N_{e}$ ) of Anatolian Merino sheep up to 400 generations ago. The distribution of $N_{e}$ values per generation was visualized by the ggplot2 package (Villanueva and Chen, 2019), implemented in the R environment (https://www.r-project.org, last access: 9 January 2025). ROH was defined to estimate the genome-wide inbreeding ( $F_{\mathrm{ROH}}$ ) value. Two statistical models, namely ROH and integrated haplotype score (iHS), were chosen to detect genomic regions under selection pressure. By running the detectRUNs (Biscarini et al., 2018) package with a consecutive-run algorithm in the R environment (https://www.r-project.org). ROH was defined according to the criteria adopted by Demir (2024b) to reveal selection signatures in Kangal Akkaraman sheep. The iHS values per SNP were calculated via the rehh package (Gautier and Vitalis, 2012) implemented in the R environment (https://www.r-project.org) after the SNPs were phased in chromosome-wise by the Beagle 5.4 software (Browning et al., 2021).

A methodology reported by Argun Karsli et al. (2024) was followed to detect outlier SNPs and gene annotation. For this purpose, the Manhattan plots for ROH and iHS values were constructed via the qqman package (Turner, 2014) implemented in the R environment (https://www.r-project.org), in which only the top 0.1 % of SNPs were considered to be under selection according to an empirical distribution. Furthermore, non-overlapping genomic intervals of 200 kb (100 kb upstream and 100 kb downstream) were screened to detect protein-coding genes via the Biomart platform (https://www.ensembl.org/info/data/biomart/index.html, last access: 9 January 2025). The effects of detected protein-coding genes on phenotype were validated via the sheep QTL database (https://www.animalgenome.org/cgi-bin/QTLdb/OA/index, last access: 9 January 2025).

## 3 Results

**Figure 1:**
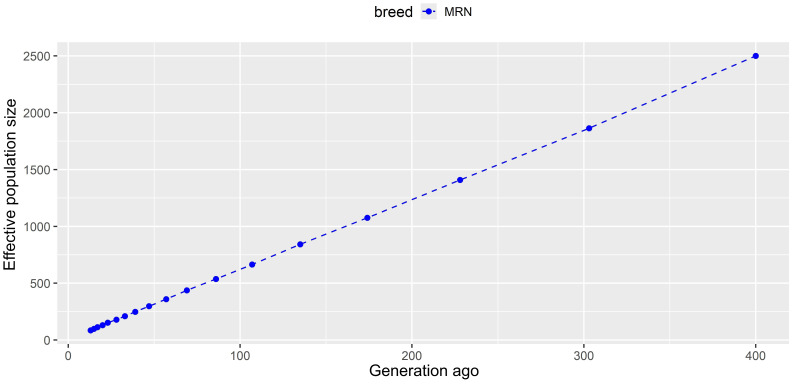
Historical changes in effective population size of Anatolian Merino sheep.

The average MAF and π values were estimated to be 0.340 and 0.235, respectively, in Anatolian Merino sheep. A higher observed heterozygosity (0.258) was detected compared to the average expected value (0.235), most probably due to the sampling strategy used, in which unrelated animals were chosen from different herds. The lowest and highest $F_{\mathrm{ROH}}$ values ranged from 0.018 in chromosome 14 to 0.047 in chromosome 6, with a mean of 0.027. The linkage-disequilibrium-based algorithm showed that effective population size decreased from one generation to another. Indeed, the current population turned out to be represented by 537, 1075, and 2500 individuals 86, 174, and 400 generations ago, respectively (Fig. 1).

A total of 1345 ROH islands, of which 1342 were clustered into 0–2 Mb based on their physical length, were observed. Only three islands turned out to be clustered into 2–4 Mb according to their physical length. Using the ROH and iHS approaches, a total of, respectively, 464 and 259 SNPs turned out to be under selection pressure (Fig. 1). Detailed information about genes under selection and their effects on phenotype can be found in a summary in the Supplement.

**Figure 2:**
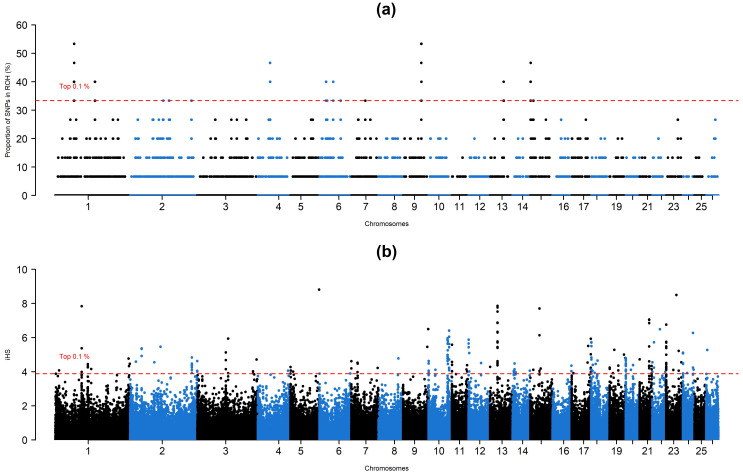
Manhattan plot for **(a)** percentage of ROH distribution and **(b)** iHS values per SNP.

In the ROH approach, detected SNPs covered 14 genomic intervals distributed over 8 chromosomes. These genomic intervals overlapped with 37 protein-coding genes, in which the lowest number of genes was observed in chromosome 7 (*TEX9*), while seven different genes were detected in chromosomes 1 (*BTBD8*, *C1orf146*, *GLMN*, *RPAP2*, *GFI1*, *EVI5*, and *ROBO1*) and 15 (*IZUMO1R*, *GPR83*, *MRE11*, *ANKRD49*, *AASDHPPT*, *KBTBD3*, and *MSANTD4*). A survey based on the sheep QTL database revealed that selection signals detected by the ROH approach overlapped with 24 QTL-related SNPs which were associated with milk content (*ROBO1*, *FSIP2*, *COBLL1*, *PTPN12*, *GSAP*, *CCDC146*, *FGL2*, *FAM185A*, *FBXL13*, *EMCN*, *DDIT4L*, *H2AZ1*, and *DNAJB14*), total muscle area (*PCDH7*), udder morphology, and immunoglobulin A level in blood (*SYBU*, *EBAG9*, *PKHD1L1*, *ENY2*, *NUDCD1*, and *TRHR*).

Regarding the iHS approach, selection signatures were detected in all chromosomes except for chromosome 25. Of these chromosomes, 3, 4, 6, 8, 9, 11, 15, 17, 20, 22, and 26 contained single genes, while selection signals were detected in 11 genes (*GRIN3A*, *RNF20*, *PGAP4*, *ALDOB*, *LOXL2*, *R3HCC1*, *CHMP7*, *RHOBTB2*, *PEBP4*, *LGSN*, and *SCYGR4*) in chromosome 2. The highest number of SNPs (35) was detected between 0–200 000 intervals of chromosome 20, which turned out to be a non-coding region according to up-to-date reference genome assembly. Selection pressure was detected at 79 genomic intervals containing 72 protein-coding genes. A total of 42 QTL-related SNPs were identified across these genomic intervals. These SNPs were previously reported to be associated with milk content (*LOXL2*, *R3HCC1*, *CHMP7*, *RHOBTB2*, *PEBP4*, *SRGAP3*, and *RAD18*), fecal egg and milk bacterial count (*LGSN* and *RUNX2*), udder morphology (*SYT1*, *GFPT2*, *MAPK9*, and *RASGEF1C*), immunoglobulin A level in blood (*RFXAP*, *SERTM1*, *BEND7*, *PRPF18*, *FRMD4A*, and *GRAMD1B*), bone density (*SDK2*), carcass traits (*MBL2*), and resistance or susceptibility to maedi-visna virus (*DLGAP1*).

## 4 Discussion

Genetic diversity parameters in native Turkish sheep breeds have mainly been assessed via microsatellite markers for the last 2 decades (Yilmaz et al., 2014; Ameur et al., 2020; Karsli et al., 2020; Kirikci et al., 2020), while SNP data obtained from NGS and array technologies are preferred to monitor genome-wide genetic variability nowadays (Ceccobelli et al., 2023; Bayraktar, 2024; Demir, 2024b; Karsli, 2024). Bayraktar (2024) reported higher observed heterozygosity (ranging from 0.348 to 0.356) compared to expected values (between 0.310 and 0.340) in three Anatolian sheep breeds known as Sakız, Norduz, and Karakaş, which were genotyped with the Illumina OvineSNP50 BeadChip. Inbreeding coefficient values, on the other hand, were declared to range from - 0.063 (Sakız) to - 0.019 (Karakaş) (Bayraktar, 2024). In another study, four native Turkish sheep breeds (Akkaraman, Güney Karaman, Karakaş, and Morkaraman) were genotyped via ddRADseq to identify genome-wide variability (Karsli, 2024). Similarly, observed heterozygosity (0.296–0.315) was reported to be higher than expected (0.287–0.299), together with negative values of the inbreeding coefficient (ranging from - 0.060 to - 0.034). The author declared that MAF values were between 0.311 in the Morkaraman breed and 0.316 in the Akkaraman and Güney Karaman breeds (Karsli, 2024). Similarly, a 50 K Ovine SNP chip-based study showed a higher observed heterozygosity (0.399) than expected (0.352) in Anatolian Merino sheep (Ceccobelli et al., 2023). On the contrary, another up-to-date study conducted by Demir (2024b) highlighted that the observed heterozygosity (0.290) was slightly lower than the expected heterozygosity (0.300), together with an MAF value of 0.320 in Kangal Akkaraman sheep, genotyped via a genotyping-by-sequencing (GBS) technique. The author indicated that the inbreeding coefficient was close to zero (0.01) in Kangal Akkaraman sheep according to the ROH-based inbreeding coefficient ( $F_{\mathrm{ROH}}$ ) (Demir, 2024b). Compared to Kangal Akkaraman sheep, a higher genomic inbreeding was detected in Anatolian Merino sheep ( $F_{\mathrm{ROH}}=0.027$ ), which may require conservation programs to prevent potential inbreeding depression and loss of genetic diversity in the future. On the other hand, higher heterozygosity values were reported by SNP array technologies (Ceccobelli et al., 2023; Bayraktar, 2024) compared to in NGS-based studies (Demir, 2024b; Karsli, 2024). These inconsistent results may be explained by differences between molecular genotyping methods in which SNP arrays are designed to detect stable variations with higher variability. On the other hand, library preparation methods such as ddRADseq and GBS allow for sequencing random reads in which variations may be lower across the population. Still, this study shows consistency with previous studies in terms of showing higher heterozygosity than the expected value (Bayraktar, 2024; Karsli, 2024). However, it seems that the Anatolian Merino breed conserves lower heterozygosity and higher inbreeding compared to other local Turkish sheep breeds. This finding is not surprising due to the fact that the Turkish Merino breed has been derived from a small population and subjected to selection practices. These kinds of breeding practices have the potential to decrease heterozygosity and increase inbreeding at the genome-wide level (Karsli and Balcıoğlu, 2019). A dramatic and continuous reduction was observed in effective population size, which was estimated to be 2500 individuals 400 generations ago, in Anatolian Merino sheep. As aforementioned, the loss in the economic value of wool production over the last 6 decades, as well as lower adaptation to environmental challenges, may lead to a decrease in the effective population size of Anatolian Merino sheep. This decreasing trend was also reported by Ceccobelli et al. (2023) via 22 682 SNPs, in which the effective size of Anatolian Merino sheep was estimated to be 105 and 332 animals 13 and 50 generations ago, respectively. This trend has been declared for numerous local sheep breeds across the world (Ablondi et al., 2020; Zhang et al., 2020; Zinovieva et al., 2020; Liu et al., 2021). For example, a GBS-based study on 22 Kangal Akkaraman sheep confirmed that the actual population was descended from 978 animals 150 generations ago (Demir, 2024b).

In this study, a total of 37 and 72 protein-coding genes were identified to be under selection pressure in Anatolian Merino sheep via ROH and iHS approaches, respectively. Similarly, a lower number of genes were detected via ROH (32 genes) compared to via iHS (136 genes) in four Anatolian sheep breeds (Argun Karsli et al., 2024). Compared to other statistical approaches, a lower number of genes under selection pressure detected via ROH were also reported in several livestock species, including some native Indian sheep (Saravanan et al., 2021) and cosmopolitan beef cattle (Moravčíková et al., 2019). As highlighted by Argun Karsli et al. (2024), these differences may occur due to the different assumptions of various statistical models in which strict criteria allow for monitoring a lower number of genomic intervals in the ROH approach. In this study, no common selection signals across protein-coding genes were detected via either the ROH or iHS approach. As mentioned by Demir et al. (2023b), this could be observed since each method scans different signals under different statistical assumptions. A total of 28 protein-coding genes detected by the iHS approach in Turkish Merino sheep were also reported to be under selection pressure in some native Turkish sheep breeds (Argun Karsli et al., 2024). Among these common genes, *R3HCC1*, *CHMP7*, *RHOBTB2*, and *GRAMD1B* were reported to be under selection pressure in all studied sheep breeds (Argun Karsli et al., 2024). According to the sheep QTL database, these genes are associated with milk content (*R3HCC1*, *CHMP7*, *RHOBTB2*) and immunoglobulin A level in blood (*GRAMD1B*). Another gene observed in this study (*LGSN*) was also reported to be under selection pressure in six native Anatolian (Anatolian Black, East Anatolian Red, South Anatolian Yellow, South Anatolian Red, Turkish Grey Steppe, and Zavot) and two cosmopolitan (Holstein Friesian and Brown Swiss) cattle breeds reared in Türkiye via the iHS approach (Demir et al., 2023b). The authors highlighted that *LGSN* plays a key role in the visual modality in humans, while it is associated with fecal egg and milk bacterial count in sheep according to the QTL database. It is known that the downregulation of the *DLG1* protein may increase the infection rate of human immunodeficiency virus (HIV) in mammals (Perugi et al., 2009), while the *DLGAP1* gene plays a key role in maintaining the regulation of the *DLG1* protein. Indeed, White et al. (2012) aimed to investigate the genetic basis of ovine lentivirus in 964 animals belonging to Rambouillet, Polypay, and Columbia sheep breeds via a genome-wide association study in which the *DLGAP1* gene turned out to be directly associated with resistance to ovine lentivirus. Significant selection signals overlapping with this gene were detected in Anatolian Merino sheep; this could be subjected to selection studies together with the *LGSN* gene to improve adaptation to environmental challenges.

## 5 Conclusions

In this study, genomic diversity and selection signals in Anatolian Merino sheep were assessed for the first time by 351 539 bi-allelic SNPs recovered from the ddRADseq technique combined with the Illumina Nova-Seq 6000 instrument. Compared to previous studies on native Turkish sheep breeds, a lower genetic variability and a higher inbreeding coefficient were detected in Anatolian Merino sheep. According to current findings, on the other hand, it can be stated that adequate genetic variability has been conserved at the genome-wide level, which is promising for sustaining current and future demands for economically important traits. However, the effective population size shows a declining trend per generation. This trend may negatively affect the agricultural sector in the future. Therefore, comprehensive and effective breeding programs should be adopted by farmers to prevent bottleneck effects in the future while periodically monitoring genetic variability to foresee future challenges. ROH and iHS approaches revealed significant selection signals in 109 protein-coding genes, most of which were directly linked to milk production. Since Merino sheep are commonly raised for wool production, selection signals are expected to occur in wool-related genes. However, as mentioned above, the declining demand for high-quality fleece in the textile sector since the 1950s has forced farmers to focus on meat and milk traits. Therefore, it is not surprising to identify selection signals in milk-related genes in Anatolian Merino sheep. This fact also confirms that selection signature analyses based on high-density SNP data are significantly efficient in revealing deeper information about the past breeding history of livestock species without requiring phenotypic records.

## Supplement

10.5194/aab-68-161-2025-supplement The supplement related to this article is available online at https://doi.org/10.5194/aab-68-161-2025-supplement.

## Data Availability

The data set used in this study is available for scientific purposes only upon request via the Material Transfer Agreement (MTA) signed by the corresponding author.
