# Supplementary material for: Genome-wide genetic characterization and selection signatures in Anatolian Merino sheep
Source: Arch Anim Breed. 2025 Feb 26;68(1):161–9. doi: 10.5194/aab-68-161-2025 (PMC13240681; doi:10.5194/aab-68-161-2025)
Supplement: The supplement related to this article is available online at https://doi.org/10.5194/aab-68-161-2025-supplement. [file aab-68-161-2025-supplement.zip › aab-68-161-2025-supplement-title-page.pdf]

Supplement of Arch. Anim. Breed., 68, 161–169, 2025
https://doi.org/10.5194/aab-68-161-2025-supplement
© Author(s) 2025. CC BY 4.0 License.

Open Access
Archives Animal Breeding

Supplement of

Genome-wide genetic characterization and selection signatures in Anatolian Merino sheep

Taki Karsli

Correspondence to: Taki Karsli (takikarsli@ogu.edu.tr)

- aab-68-161-2025-supplement-title-page.pdf
- Supplementary_File.xlsx

The copyright of individual parts of the supplement might differ from the article licence.
